# A Diagnostic Challenge of Facial Nerve Palsy and Hearing Loss With Bilateral Otomastoiditis in an Elderly Patient

**DOI:** 10.7759/cureus.103256

**Published:** 2026-02-09

**Authors:** Khushbakht Subhani, Hesham Ibrahim

**Affiliations:** 1 Emergency Department, Hampshire Hospitals NHS Foundation Trust, Basingstoke, GBR

**Keywords:** facial nerve palsy, otomastoiditis, ramsay hunt syndrome, sensorineural hearing loss, temporal bone ct, zoster sine herpete

## Abstract

Facial nerve palsy in older adults is commonly idiopathic or viral in origin, but secondary otologic and neurological causes must be carefully excluded, particularly when associated with hearing loss. We report a diagnostically challenging case of a 79-year-old woman who presented with acute right lower motor neuron facial palsy and new-onset hearing impairment, without otalgia, rash, or otorrhoea. Examination confirmed severe right-sided facial weakness, and initial imaging with computed tomography (CT) of the head and temporal bones demonstrated bilateral mastoid and middle ear opacification without bony erosion or intracranial extension. Despite these findings, the clinical presentation was not consistent with otogenic facial palsy.

Given the combination of complete unilateral facial paralysis, hearing impairment, and absence of vesicular eruption, Ramsay Hunt syndrome presenting as zoster sine herpete was suspected. The patient was transferred to a tertiary otolaryngology centre and commenced on antiviral therapy and systemic corticosteroids. Subsequent specialist follow-up identified bilateral mixed hearing loss with a left-sided conductive component due to middle ear effusion, which did not anatomically or temporally correlate with the acute right-sided facial palsy. The patient experienced gradual clinical improvement.

This case highlights the diagnostic complexity that arises when imaging findings mimic otologic infection but the clinical picture supports viral neuritis. It underscores the importance of cautious interpretation of radiologic findings and early recognition of Ramsay Hunt syndrome even in the absence of vesicular rash, as timely initiation of antiviral and corticosteroid therapy may improve neurological outcomes.

## Introduction

Facial nerve palsy is most commonly idiopathic; however, secondary causes such as infection, inflammation, and viral reactivation must be carefully excluded, particularly in older adults. Ramsay Hunt syndrome, caused by reactivation of varicella-zoster virus, classically presents with peripheral facial paralysis and vesicular eruptions. Notably, up to one-third of cases may occur without rash (zoster sine herpete), making diagnosis more challenging and reliant on clinical pattern recognition [1,2]. Early initiation of antiviral therapy combined with corticosteroids has been shown to improve recovery outcomes in Ramsay Hunt syndrome [3].

Otitis-related facial palsy has become uncommon in the antibiotic era but may still occur due to inflammatory extension to the facial nerve canal, most often in the context of acute otitis media or mastoiditis [4,5]. Distinguishing viral neuritis from otogenic pathology can be difficult when clinical and radiological features overlap. This report describes an elderly patient with acute unilateral facial palsy and hearing loss in whom imaging demonstrated bilateral otomastoid inflammatory changes despite the absence of typical otologic symptoms, highlighting the diagnostic challenges in differentiating coincidental imaging findings from the primary neurological process.

## Case presentation

A 79-year-old woman presented to the emergency department with a three-day history of right-sided facial weakness and new difficulty hearing on the same side. Communication required a raised voice, which was entirely new. She denied fever, otalgia, otorrhoea, dizziness, vesicular rash, tinnitus, hyperacusis, altered taste sensation, vertigo, recent trauma, or constitutional symptoms. She reported only a mild “funny feeling” around the left ear. There were no visual changes, speech disturbance, limb weakness, or gait abnormality.

Her medical history included hypertension and previous excision of a basal cell carcinoma beneath the right eye. Medications included losartan, lercanidipine, sertraline, omeprazole, simvastatin, aspirin, alendronic acid, and Adcal-D3. She was independent in activities of daily living and had no smoking or alcohol history.

On examination, she was afebrile and clinically stable. Neurological examination demonstrated complete right lower motor neuron facial palsy (House-Brackmann grade VI) with loss of forehead creases, incomplete eye closure, and facial asymmetry at rest. Facial sensation was intact. Hearing was subjectively reduced on the right; bedside tuning fork testing (Weber/Rinne) was not performed at the initial emergency assessment. The remainder of the cranial nerve examination was normal, with no cerebellar signs. Otoscopy revealed impacted cerumen in the right external auditory canal, initially limiting visualization of the tympanic membrane. The cerumen was subsequently removed, and repeat examination demonstrated a normal appearing right tympanic membrane with no evidence of erythema, effusion, or perforation. Mild erythema of the left tympanic membrane was noted incidentally.

**Figure 1:**
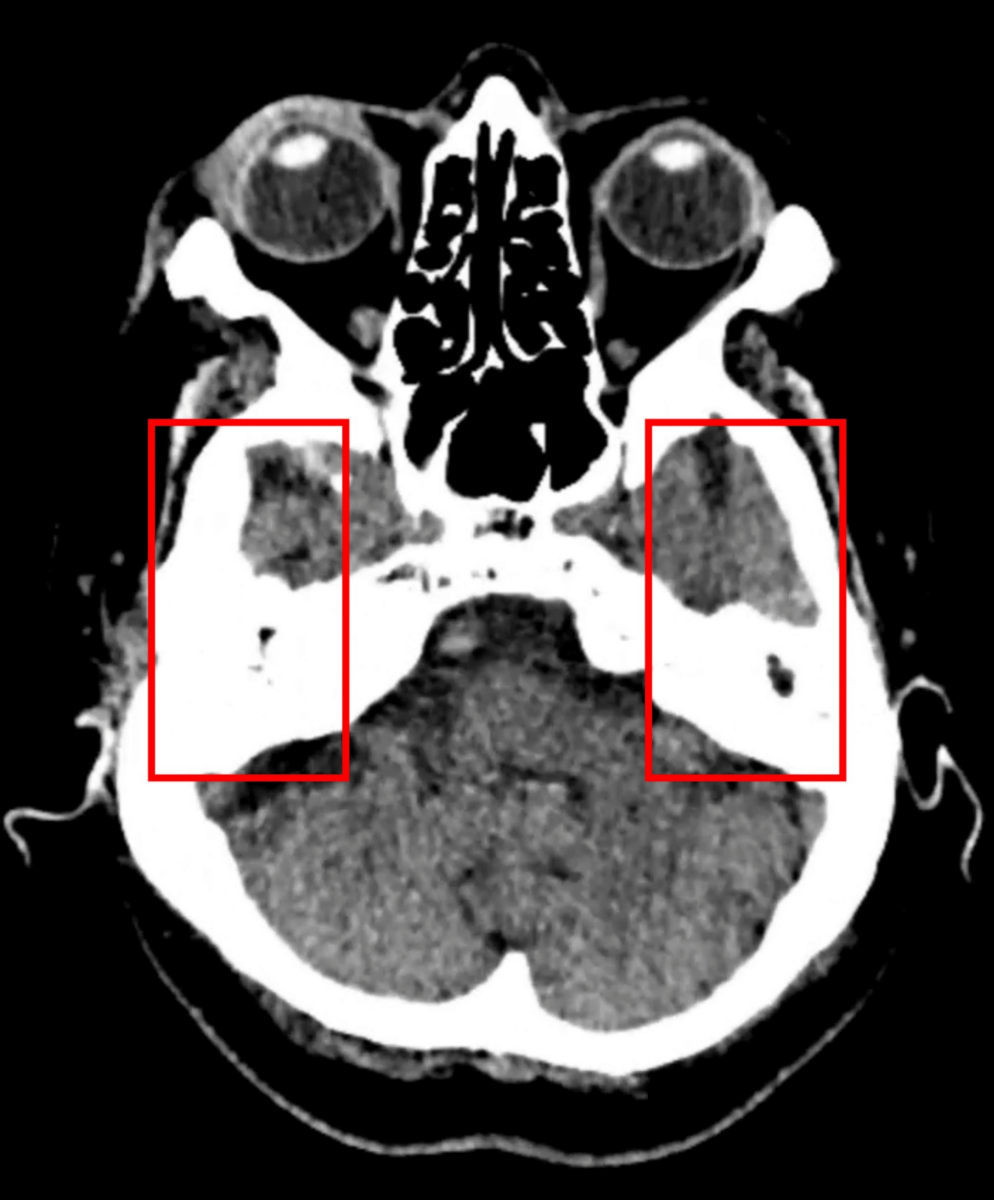
Axial CT showing bilateral mastoid opacification Axial CT demonstrating complete soft tissue opacification of both mastoid air cells without bony erosion, consistent with bilateral otomastoid inflammatory change.

Blood tests demonstrated an elevated C-reactive protein of 32 mg/L (normal <5 mg/L) and a normal leukocyte count of 6.6 × 10⁹/L (range 4-11 × 10⁹/L). Electrolytes and renal function were within normal limits. Mild biochemical abnormalities included an alkaline phosphatase of 131 U/L (range 30-130 U/L), a reduced albumin of 31 g/L (range 34-50 g/L), and lymphocytes of 0.92 × 10⁹/L (range 1.3-4 × 10⁹/L). Liver enzymes were within normal limits. Computed tomography (CT) of the head and temporal bones demonstrated bilateral opacification of the mastoid air cells without bony erosion, sigmoid sinus thrombosis, intracranial abscess, or mass lesion (Figure 1). No acute territorial infarction was identified. These findings were not felt to adequately explain the severity or laterality of the facial nerve dysfunction, supporting consideration of a primary neural process. These findings were discussed with the ENT team and were not felt to represent acute coalescent mastoiditis.

**Figure 2:**
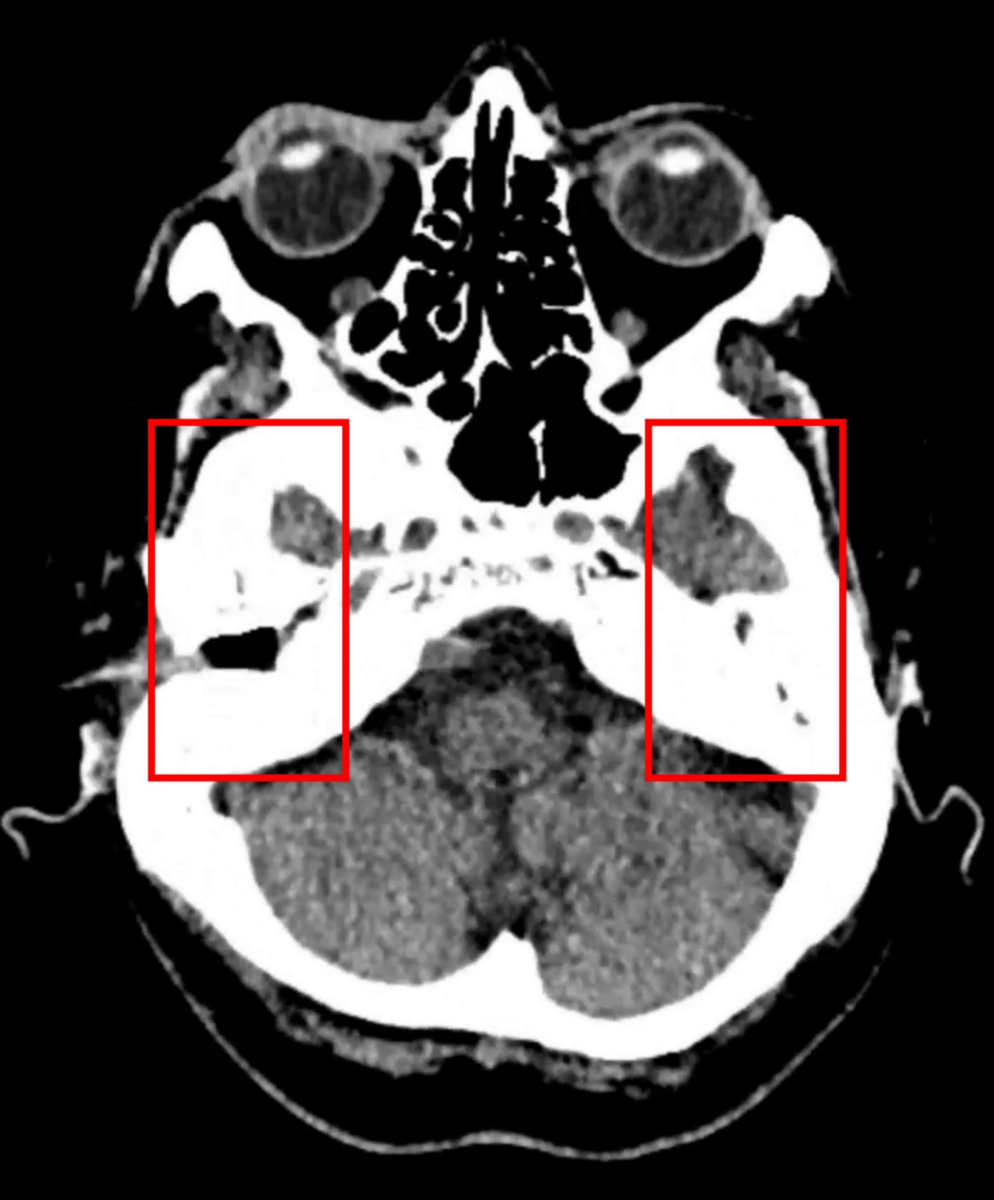
Axial CT showing middle ear cavity opacification Opacification of the middle ear cavities, more marked on the right, without evidence of coalescent mastoiditis or ossicular erosion.

**Figure 3:**
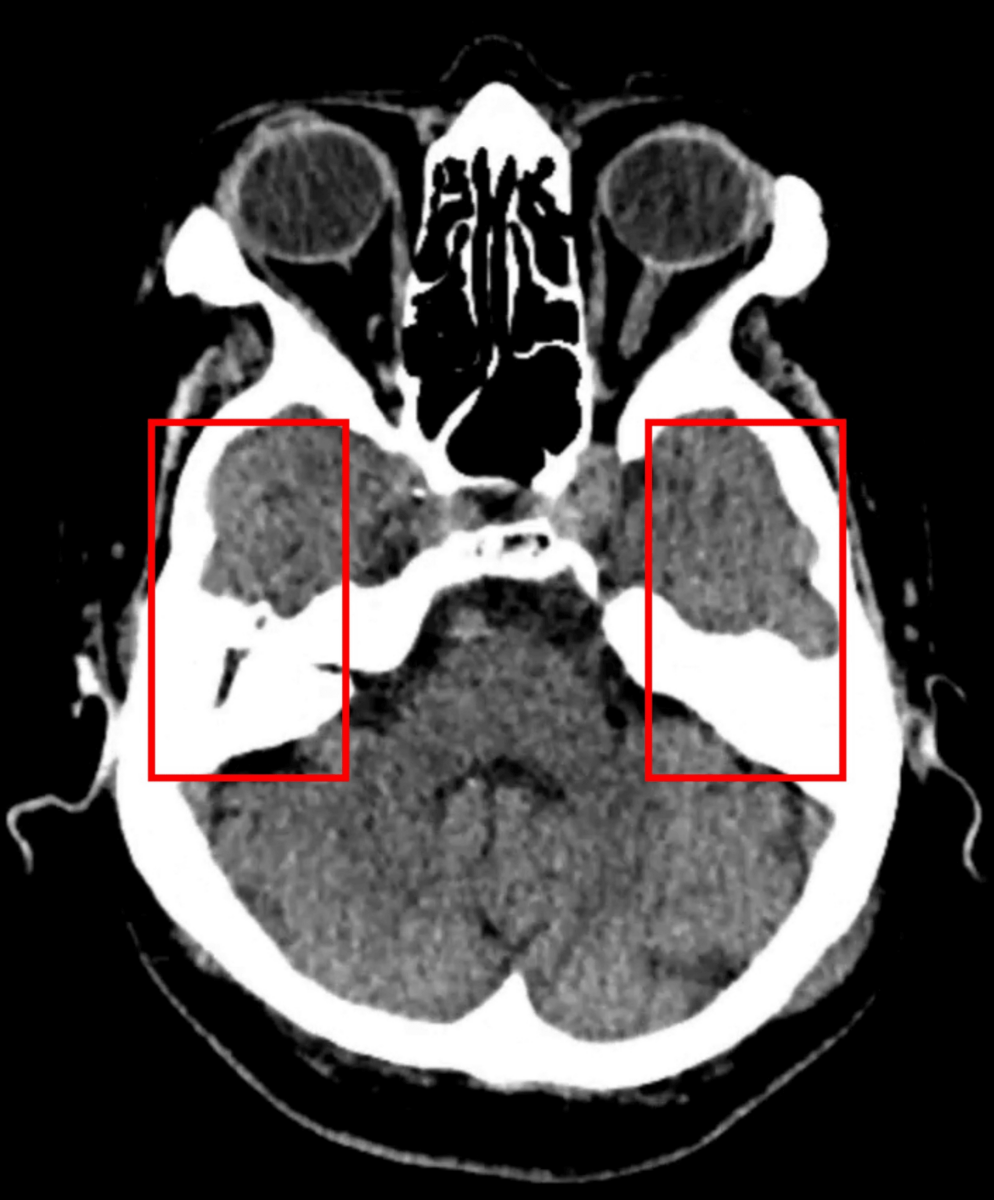
Axial CT demonstrating intact ossicular chain The ossicular chain appears intact bilaterally with preserved bony margins, making erosive infectious disease less likely.

**Figure 4:**
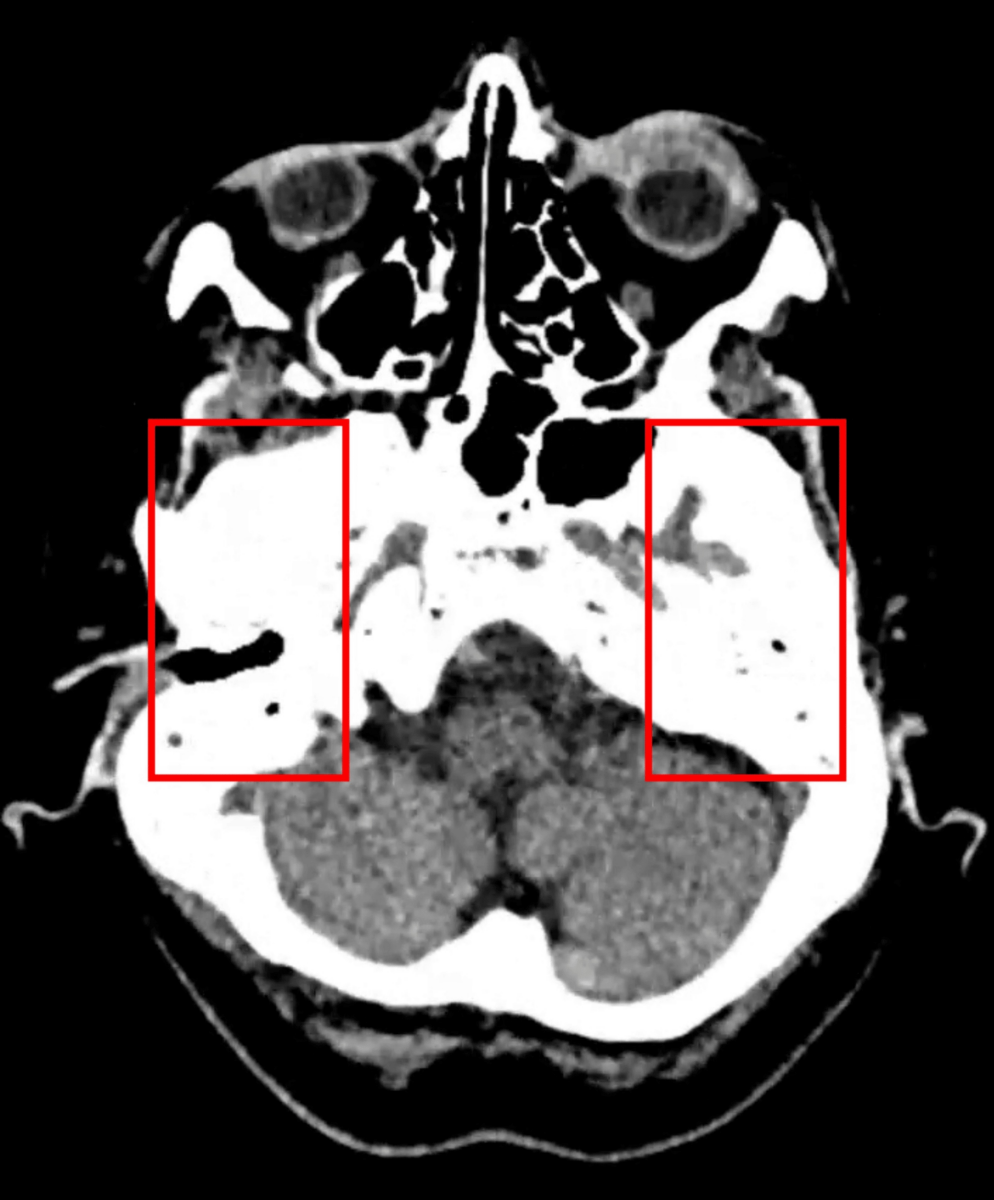
Axial CT showing soft tissue density in the external auditory canal Mild soft tissue debris is noted within the right external auditory canal, likely representing impacted wax or local inflammatory tissue.

Additional CT slices demonstrated opacification of the middle ear cavities (Figure 2) with preservation of the ossicular chain bilaterally (Figure 3), making erosive otologic infection less likely. Soft tissue density was also noted within the right external auditory canal (Figure 4), consistent with cerumen or non-specific debris.

Given the acute onset of complete unilateral facial paralysis with hearing impairment in the absence of pain, discharge, or systemic infection, idiopathic Bell’s palsy was considered unlikely. The imaging findings were not felt sufficient to explain the severity or laterality of facial nerve dysfunction, raising suspicion for viral neuritis, most consistent with Ramsay Hunt syndrome presenting as zoster sine herpete.

The patient was transferred to a tertiary hospital with on-call otolaryngology services. Although full inpatient records from the receiving hospital were unavailable, follow-up with the patient confirmed that she was commenced on oral acyclovir and systemic corticosteroids (prednisolone) within the first few days of symptom onset. Antibiotics were not initiated due to the absence of clinical features of bacterial mastoiditis.

On subsequent outpatient ENT review, both external auditory canals were clear with no evidence of wax or active infection. Otoscopy demonstrated a left-sided middle ear effusion. Formal audiometry revealed bilateral profound hearing loss, worse on the left, consistent with presbycusis with an additional conductive component; overall, this was classified as mixed hearing loss. Tympanometry demonstrated a type B trace on the left and a type A trace on the right. Management included intranasal decongestants (xylometazoline for seven days), intranasal corticosteroid drops (betamethasone), and autoinflation using an Otovent balloon. The patient reported gradual improvement in facial nerve function and was referred for facial physiotherapy and routine ENT follow-up.

## Discussion

This case illustrates the diagnostic complexity encountered when clinical and imaging findings overlap between viral neuritis and otologic inflammatory disease. Ramsay Hunt syndrome is a recognized cause of facial nerve palsy in older adults and may present without vesicular rash in cases of zoster sine herpete, delaying diagnosis and treatment [1,2]. Early initiation of antiviral therapy combined with corticosteroids is associated with improved neurological outcomes [3]. Zoster sine herpete has been reported to comprise up to 30% of Ramsay Hunt syndrome cases, underscoring the need for clinical vigilance when acute facial palsy is accompanied by auditory symptoms despite absent vesicles [6].

Facial nerve palsy secondary to otologic disease is now rare in the antibiotic era and is typically associated with otalgia, otorrhoea, fever, or conductive hearing loss [4]. In this case, CT imaging demonstrated bilateral mastoid and middle ear opacification without erosive change or intracranial complications. In the absence of supportive clinical features, these findings were interpreted as reactive or incidental inflammatory change rather than acute infection. This interpretation is supported by evidence demonstrating poor correlation between mastoid opacification on CT and true clinical mastoiditis and recommending cautious reporting when bony erosion is absent [7].

Imaging must be interpreted within the clinical context. CT is frequently used acutely to exclude hemorrhage, mass lesions, skull base pathology, and erosive temporal bone disease, but it has limited sensitivity for facial nerve enhancement, internal auditory canal and cerebellopontine angle lesions, labyrinthine inflammation, and subtle brainstem pathology. MRI with gadolinium is preferred when these etiologies are suspected [8,9]. In this case, MRI was considered but not obtained at the referring hospital due to limited immediate availability and the decision to expedite transfer for specialist otolaryngology review. The absence of focal neurological deficits or brainstem signs, non-erosive CT findings, and subsequent clinical improvement following antiviral and corticosteroid therapy supported a viral neuritis process.

Later identification of mixed hearing loss with a left-sided conductive component reflected co-existing age-related and middle ear pathology and did not anatomically or temporally correlate with the acute right-sided facial palsy, further supporting a primary neurological rather than otogenic aetiology.

## Conclusions

Acute facial nerve palsy with associated hearing loss in older adults requires careful evaluation beyond idiopathic causes. Ramsay Hunt syndrome should be considered even in the absence of vesicular rash, as zoster sine herpete is a recognized presentation of varicella-zoster virus reactivation. Radiologic findings suggestive of otomastoiditis should be interpreted cautiously and correlated with clinical features, as non-erosive mastoid opacification may be incidental. Early recognition and timely initiation of antiviral and corticosteroid therapy may improve neurological recovery and functional outcomes.
